# Confinement of Suspension-Cultured Cells in Polyethylene Glycol/Polyethylene Oxide-Albumin Aqueous Two-Phase Systems

**DOI:** 10.3389/fchem.2019.00441

**Published:** 2019-06-18

**Authors:** Alyne G. Teixeira, Alex Kleinman, Rishima Agarwal, Nicky W. Tam, Jun Wang, John P. Frampton

**Affiliations:** ^1^School of Biomedical Engineering, Dalhousie University, Halifax, NS, Canada; ^2^Pomona College, Claremont, CA, United States; ^3^Department of Microbiology and Immunology, Dalhousie University, Halifax, NS, Canada; ^4^Canadian Center for Vaccinology, IWK Health Centre, Halifax, NS, Canada

**Keywords:** aqueous two-phase systems, cell partitioning, polyethylene glycol, polyethylene oxide, bovine serum albumin, immune cells, ELISpot

## Abstract

Aqueous two-phase systems (ATPSs) have numerous applications in separation science, and more recently, in bioassays enabled by the solution micropatterning of cells. The most frequently used ATPS in these applications is the polyethylene glycol (PEG)-dextran (Dex) system, as the polymers that form this ATPS have been extensively characterized in terms of their physicochemical properties. However, in addition to this well-known system, there exist many other ATPSs with properties that may be exploited to improve upon the PEG-dextran system for specific applications. One of these underexplored systems is the ATPS formed from PEG/polyethylene oxide (PEO) and albumin. In this article, we characterize the phase separation of PEG (35 kDa) and polyethylene oxide (PEO) (200, 900, and 4,000 kDa) with bovine serum albumin (BSA). We describe the microscopic emulsion behavior of these systems in the presence of NaCl and compounds (NaHCO_3_, NaH_2_PO_4_, and HEPES) commonly used in buffer solutions and cell culture media. We further demonstrate that PEG- and PEO-albumin systems can be used in place of the PEG-dextran system for confinement of suspension-cultured cells (Jurkat T cells and RPMI-8226 B cells). Cell viability and morphology are examined for various polymer formulations relative to the commonly used PEG 35 kDa-Dex 500 kDa system and polymer-free cell culture medium. In addition, we examine cell activation for various phase-separating medium components by measuring IL-2 and IL-6 secretion. We demonstrate that we can confine immune cells and cytokines in the PEG-BSA system, and that this system can be employed to screen immune responses by enzyme-linked immunospot (ELISpot) assay. This new system represents a promising ATPS formulation for applications where low levels of baseline cell activation are required, for instance, when culturing immune cells.

## Introduction

Aqueous two-phase systems (ATPSs) form when two incompatible polymers, a polymer and a salt, or a polymer and a surfactant exceed threshold concentrations in a common aqueous solvent (Albertsson, 1971; Akbulut et al., 2012). Each system can be characterized by a phase diagram that displays the concentrations at which the solutes phase-separate. A curved line called the binodal separates two areas: compositions represented by points above the binodal that give rise to phase separation and compositions represented by points below the binodal that mix to form a single phase (Albertsson, 1971; Teixeira et al., 2018). Phase separation can be influenced by physiochemical properties such as the polymer molecular weight (MW) and concentration, temperature, pH, and ionic concentration (Diamond and Hsu, 1992; Kaul, 2000; Cabral, 2007). For instance, higher MW polymers require lower concentrations for phase separation (Albertsson, 1971). In addition, the binodal curve delineating sub-critical concentrations from supercritical concentrations becomes more asymmetrical with increasing difference in MW between the two polymers (Albertsson, 1971). The system properties also influence the distribution of cells and biomolecules between the two phases.

ATPSs provide a mild non-toxic and non-denaturing environment for the partitioning of cells and biomolecules with applications ranging from isolation and recovery of antibodies (Muendges et al., 2015; Ferreira et al., 2016), proteins (da Silva et al., 2015; de Albuquerque Wanderley et al., 2017), virus-like particles (Jacinto et al., 2015; Ladd Effio et al., 2015), antibiotics (Pereira et al., 2012; Marques et al., 2013), (Johansson et al., 2012; Wiendahl et al., 2012), cells (Cabral, 2007; Zimmermann et al., 2016), extracellular vesicles (Shin et al., 2015), and hormones (He et al., 2005; Li et al., 2015) to solution micropatterning of cancer cells, hepatocytes and keratinocytes (Frampton et al., 2013; Agarwal et al., 2017). The most extensively used ATPS for these applications is the polyethylene glycol (PEG)-dextran (Dex) system. The cost-effectiveness and lack of acute cytotoxicity of the PEG-Dex system allows the precise positioning of cells by dispensing single-microliter or sub-microliter droplets of a cell-laden Dex solution within a PEG solution. The interfacial tension formed between the two polymer solutions confines the cells in the Dex droplets, where they can then adhere to the culture substrate. For adherent cells, the polymer solutions are removed once the cells attach to the substrate. However, confinement of suspension-cultured cells in an ATPS poses a challenge, as the polymers must remain in place for longer periods of time to maintain cell confinement. For culture periods longer than 24 h, cytotoxicity and cellular activation can vary by polymer type and concentration.

Here, we present an under-explored system for confinement of suspension-cultured cells that utilizes a PEG/polyethylene oxide (PEO) and bovine serum albumin (BSA) ATPS. Our objectives were to characterize phase separation of this novel system, evaluate the effects of PEG, PEO, and BSA on immune cell cytotoxicity and activation, and to demonstrate the utility of the PEG-BSA system for confinement of immune cells and reagents to reduce assay costs associated with the gold-standard enzyme linked immunospot (ELISpot) assay for measuring cytokine secretion from suspension-cultured cells. PEG and PEO have the same chemical structure. Industry conventions refer to low MW ethylene glycol polymers (less than ~35,000 Da) as PEG, while ethylene glycol polymers greater than ~35,000 Da are referred to as PEO (Wang et al., 2000). PEG and PEO are applied extensively in the food, cosmetic, and pharmaceutical industries. PEG and PEO are also widely used as biomaterials to alter surface properties to repel proteins (Lee et al., 1995), and have been applied for biomedical applications such as coating of medical devices and nanoparticles (Aqil et al., 2008). As a major amphiphilic plasma protein, albumin has been used as an essential nutrient for cell culture. Albumin has also been widely used in cell culture due to its role as an antioxidant and carrier of important biomolecules (Francis, 2010). ATPSs have been used for the extraction of BSA from biological fluids (Pei et al., 2009; Lu et al., 2010). However, to the best of our knowledge, this article is the first to use BSA as an ATPS-forming polymer. We demonstrate that PEG- and PEO-albumin systems are promising ATPS formulations for confinement of suspension-cultured cells such as T cells and B cells that require low levels of baseline cell activation. This ATPS-mediated technique enables T cell and B cell culture over the course of 72 h with minimal activation, as monitored by IL-2 and IL-6 secretion.

## Materials and Methods

### PEG- and PEO-BSA Binodals

Unless otherwise noted, all polymer solutions are reported as percent weight solutions. Stock solutions of 30% BSA (Sigma-Aldrich), 20% PEG 35 kDa (Sigma-Aldrich), 10% PEO 200 kDa, 3% PEO 900 kDa, and 2.0% PEO 4,000 kDa (Dow Chemical) were dissolved in Dulbecco's Modified Eagle's Medium (DMEM; VWR). PEO solutions were centrifuged to sediment and remove silica particles introduced by the manufacturer. Concentrated stock solutions of BSA, PEG, and PEO were introduced to 96-well plates and mixed to form super-critical emulsions. Each super-critical concentration was then titrated with polymer-free culture medium until phase separation was no longer observed using methods similar to those reported previously for binodal determination in 96-well plates (Ruthven et al., 2017). The final BSA, PEG, and PEO concentrations were determined according to

$$\begin{array}{l} C_{i}\left(V_{i}\right)=C_{f}\left(V_{f}\right), \end{array}$$

where *C*_*i*_ refers to the initial concentration,*V*_*i*_ refers to the initial volume, *C*_*f*_ refers to the final concentration and *V*_*f*_ refers to the final volume. A Nikon Eclipse Ti microscope equipped with a 10x objective lens was used to observe microscopic emulsion characteristics.

### Microdroplet Characterization

Equilibrated ATPSs composed of 15% BSA and either 7% PEG 35 kDa, 4% PEO 200 kDa, 1.5% PEO 900 kDa, or 1.5% PEO 4,000 kDa were used to characterize the stability of dispensed microdroplets. PEG- and PEO-BSA systems were centrifuged at 3,000 rcf to separate the phases for collection. Once collected, the top and bottom phases were centrifuged again at 3,000 rcf to allow removal of traces of the other phase transferred during collection. For each system, a 1 μL droplet of BSA-rich bottom phase was added to 500 μL of the PEG- or PEO- rich top phase. After 20 min (to allow droplet stabilization), a Nikon Eclipse Ti microscope equipped with a 2x objective lens was used to observe the droplets.

### Effects of Salt on Phase Separation

Solutions of 15% BSA and 1.5% PEO 900 kDa were prepared in distilled, de-ionized water containing the following salts: sodium bicarbonate (Fisher Scientific), HEPES (Sigma-Aldrich), sodium phosphate monobasic (Sigma-Aldrich) and sodium chloride (Fisher Scientific). Salts were tested at the following concentrations: 2, 4, 6, and 8 g/L. Microscopic emulsion characteristics in the presence of each salt were observed using a 10x objective lens on a Nikon Eclipse Ti microscope to determine the effects that individual medium components had on phase separation without extensively characterizing binodal curves for each system.

### Jurkat T Cell Culture

Jurkat T cells, clone E6-1 (ATCC: TIB-152), were cultured in a humidified incubator at 37°C under 5% CO_2_ in RPMI 1640 medium (VWR) supplemented with 10% fetal bovine serum (FBS) and 1% antibiotics. Medium was replaced every 2–3 days and cell density was maintained below 1 × 10^6^ cells/mL for cell propagation. Three types of plates were examined for suspension cell confinement in the PEG-BSA system: flat-bottom, round-bottom and V-bottom. Jurkat T cells were confined in the BSA phase (bottom). The BSA-phase was labeled with FITC-conjugated Dex and the cells were labeled with CellTracker Red™ CMTPX (Life Technologies) to aid in visualization. Cells cultured in the PEG-BSA system were observed using a 4x objective lens on a Nikon Eclipse Ti microscope.

### RPMI-8226 B Cell Culture

RPMI-8226 B cells (ATCC: CCL-155), were cultured in a humidified incubator at 37°C under 5% CO_2_ in RPMI 1640 medium (VWR) supplemented with 10% fetal bovine serum (FBS) and 1% antibiotics. Medium was replaced every 2–3 days and cell density was maintained below 1 × 10^6^ cells/mL for cell propagation.

### Cell Viability Assessment

Cells were seeded in 96-well culture plates at a density of 5 × 10^3^ cells per well. The cells were then incubated for 72 h in 100 μL of either the individual filter-sterilized polymer solutions or BSA at concentrations exceeding those required for phase-separation. The following polymer/BSA concentrations in RPMI 1640 medium supplemented with 10% FBS and 1% antibiotics were examined: 7% PEG 35 kDa, 2% PEO 200 kDa, 0.5% PEO 900 kDa, 0.1% PEO 4,000 kDa, 7% Dex 500 kDa (T500; Pharmacosmos), and 10% BSA. Several lots of BSA were examined including BSA (Sigma-Aldrich cat # A7906), Albumax™ I (Gibco cat # 11020-021), HyClone (GE Life Sciences cat # SH30574.02) and Cellect™ Bovine Albumin Low IgG (MP Biomedicals cat # 180576). Cells were cultured in these solutions in a humidified incubator at 37°C under 5% CO_2_ for 72 h prior to viability assessment.

Cell viability in the presence of individual polymers was assessed by live/dead staining with Calcein-AM (C-AM, Biotium) and Propidium Iodide (PI, Sigma-Aldrich). C-AM can enter live cells, where it is hydrolyzed in the cytoplasm to calcein, producing a fluorescent signal at 530 nm upon excitation with blue light (Weston and Parish, 1990). PI can only enter dead cells, where it subsequently binds to nucleic acids, primarily in the nucleus (Suzuki et al., 1997). Thus, C-AM was used to identify live cells (shown in green), whereas PI was used to identify dead cells (shown in red). A C-AM/PI stock solution (25 μM C-AM/25 μM PI) was prepared in RPMI 1640 medium. A volume of 12 μL of the stock solution was added to each well to achieve a final concentration of 3 μM for each dye. Cells were incubated in the presence of the dyes at 37°C under 5% CO_2_ for 20 min. The cells were observed by epifluorescence microscopy using a Nikon Eclipse Ti Microscope. Images were processed using ImageJ to subtract background and adjust brightness.

### Jurkat T Cell Activation

Jurkat T cells were seeded at 5 × 10^5^ cells/well in 24-well plates. The cells were stimulated with phorbol-12-myristate-13-acetate (PMA) and 300 ng/mL ionomycin. To determine the best PMA concentration to stimulate the cells as a positive control for cell activation, the following PMA concentrations were tested in culture medium: 25, 50, 100, and 200 ng/mL. Supernatants from PMA/ionomycin-stimulated cells were collected at three time points: 6-, 12- and 24-h post-stimulation. To assess Jurkat T cell activation in the presence of polymers/BSA, cells were seeded in 24-well culture plates at a density of 5 × 10^5^ cells/well in the following polymer and BSA solutions in RPMI 1640 medium supplemented with 10% FBS and 1% antibiotics: 7% PEG 35 kDa, 7% Dex 500 kDa, 10% BSA Sigma, 10% BSA Albumax, and 10% BSA HyClone. After 6, 12, and 24 h, supernatants were collected to measure IL-2 secretion.

### RPMI-8226 B Cell Activation

RPMI-8226 B cells were seeded at 5 × 10^5^ cells/well in 24-well plates. The cells were stimulated with lipopolysaccharide (LPS) from *Salmonella enterica serotype minnesota* (Sigma-Aldrich). To determine the best LPS concentration to stimulate the cells as a positive control for cell activation, the following LPS concentrations were tested in culture medium: 50, 100, and 200 ng/mL. Supernatants from LPS-stimulated cells were collected at two time points: 24- and 48-h post-stimulation. To assess RPMI-8226 B cell activation in the presence of polymers/BSA, cells were seeded in 24-well culture plates at a density of 5 × 10^5^ per well in the following polymer and BSA solutions in RPMI 1640 medium supplemented with 10% FBS and 1% antibiotics: 7% PEG 35 kDa, 7% Dex 500 kDa, and 10% BSA Albumax. After 24 h, supernatants were collected to measure IL-6 secretion.

### Measurement of IL-2 and IL-6 Secretion

Concentrations of IL-2 and IL-6 in the supernatants were determined by enzyme-linked immunosorbent assay (ELISA) according to the manufacturer's instructions (R&D Systems cat # S2050 and cat # DY206) using antibody concentrations recommended by the manufacturer. Briefly, 96-well microplates were coated overnight with capture antibodies diluted in phosphate buffered saline (PBS). The next day, the plates were washed three times in PBS containing 0.2% Tween 20 (PBST) and blocked for 1 h with 1% BSA in PBS. After blocking, the plates were washed three times in PBST and incubated with the samples and recombinant protein standards for 2 h. Next, the plates were washed three times in PBST and incubated for 2 h with detection antibodies. The plates were then washed three times in PBST and incubated with streptavidin-conjugated horseradish peroxidase (HRP) for 20 min. Finally, the plates were washed three times in PBST and incubated with SuperSignal ELISA Pico Chemiluminescent Substrate (Thermo Fisher Scientific) for 10 min. All incubation and wash steps were performed at room temperature. Luminescence was measured using a FilterMax F5 microplate reader. Unknown values were determined by extrapolation from a four-parameter logistic standard curve.

### Confinement of Cells in the PEG-BSA System for ELISpot Assay

To demonstrate the utility of the PEG-BSA (Albumax) system, an optimized formulation was used to confine immune cells and reduce reagent consumption in the ELISpot assay. Briefly, 96-well ELISpot plates (Millipore Sigma) were coated with a working solution of 10 μg/mL IL-6 capture antibody from R&D Systems cat # DY206 diluted in PBS. The plates were stored at 4°C overnight. The next day, the plates were washed with sterile water and blocked for 1 h with complete RPMI medium for 2 h. After blocking, the medium was removed, and RPMI-8226 B cells were added to each well at various cell seeding densities. To stimulate the RPMI-8226 B cells, a final concentration of 100 ng/mL of LPS was added to the 10% BSA solution. Next, a 20 μL volume of the mixture was placed in each well of the ELISpot 96-well plate and covered with 80 μL of 7% PEG solution. A conventional ELISpot assay without cell confinement in the PEG-BSA system was performed in parallel as described above, with cells and LPS mixed in RPMI medium and 100 μL of the mixture added to each well. Following 24 h of culture for both the ATPS-based assay and the conventual assay, the plates were washed with PBS containing 0.01% Tween 20 and incubated with the IL-6 detection antibody (0.1 μg/mL antibody from R&D Systems cat # DY206) at 37°C for 2 h. The plates were then washed with PBS containing 0.01% Tween 20 and incubated with streptavidin-alkaline phosphatase (Biotium) (1:1000 dilution in sterile PBS) for 45 min. Finally, the plates were washed with PBS containing 0.01% Tween 20, followed by three washes with PBS, and incubated with 100 μL of BCIP/NBT substrate (Sigma-Aldrich) per well for 5–10 min. Spot development was stopped by extensive washing under running tap water. Plates were left to dry overnight in the dark. IL-6 producing cell spots were counted with an ImmunoSpot S6 (Cellular Technology Limited).

### Measurement of IL-6 Confinement

IL-6 confinement was examined for two ATPS formulations: 7% PEG 35 kDa-10% BSA (Albumax) and 5% PEG 35 kDa-6.4% Dex 500 kDa. Both systems were formed in complete RPMI 1640 medium. A total of 500 pg/mL of recombinant IL-6 was added to the bottom phase of each system (i.e., in either BSA or Dex). A 50 μL volume of each bottom phase solution was added to a 200 μL microtube and covered with a 150 μL layer of either 7% PEG for the PEG-BSA system or 5% PEG for the PEG-Dex system. Both systems were left to achieve thermodynamic equilibrium at room temperature, and the phases were collected separately at three time points: 2, 24, and 48 h. Three replicates were used for each system. Concentrations of recombinant IL-6 in the respective phases of the PEG-BSA and PEG-Dex systems were measured by ELISA according to the manufacturer's instructions (R&D Systems cat # DY206) using the same ELISA procedure described above.

### Statistical Analysis

Kruskal-Wallis one-way analysis of variance (ANOVA) and Tukey multiple comparison tests were conducted to compare the effects of phase-separating solutions on cell viability over 72 h. Kruskal-Wallis ANOVA and Tukey multiple comparison tests were performed to compare the effects of PMA and LPS concentrations on cell activation. Kruskal-Wallis ANOVA and Dunn's multiple comparison tests were performed to compare the effects of phase-separating solutions on IL-2 and IL-6 secretion by Jurkat T cells and RPMI-8226 B cells, respectively, over 24 h. One-way ANOVA and Tukey multiple comparison tests were conducted to compare IL-6 confinement ratios over time, and the number of IL-6 secreting cell spots as a function of cell density. Data are represented as mean values + standard deviations. Statistical significance was defined as ^*^p < 0.05.

## Results

**Figure 1:**
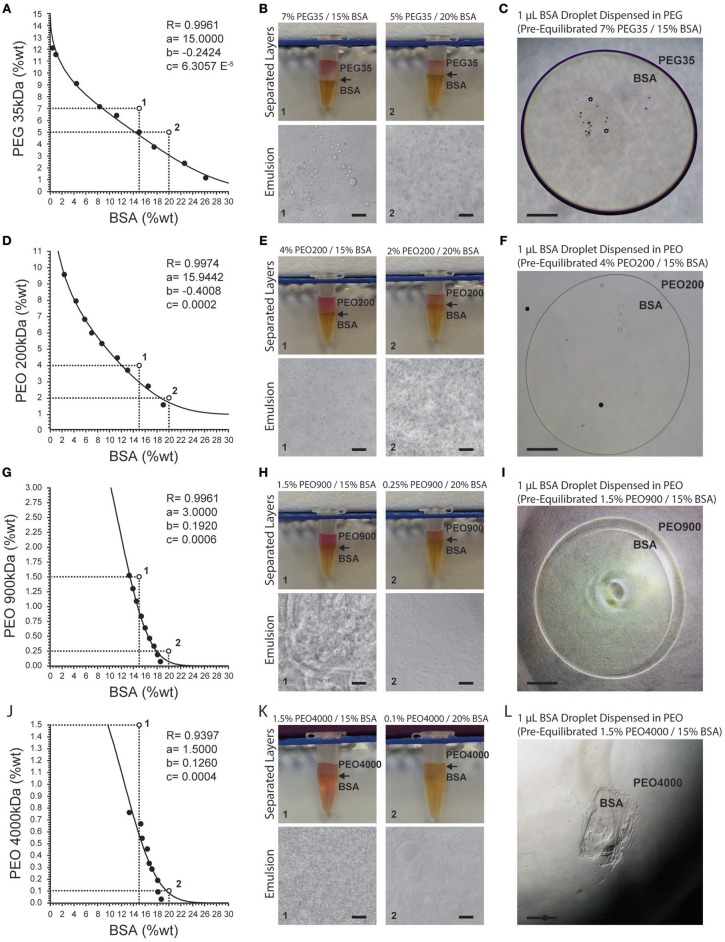
Binodal curves for PEG- and PEO-BSA systems. **(A)** PEG 35 kDa-BSA, **(D)** PEO 200 kDA-BSA, **(G)** PEO 900 kDa-BSA and **(J)** PEO 4000 kDa-BSA. R is the correlation coefficient. Fitting parameters are denoted by a, b, and c. Points 1 and 2 correspond to formulations shown in images to the right. **(B**,**E**,**H**,**K)** show macroscopic and microscopic images of PEG, PEO, and BSA at different concentrations indicated by numbered points on the binodal graphs. Arrows indicate the location of the interface between the phases. **(C,F**,**I,L)** show microscopic images of pre-equilibrated BSA droplets dispensed in pre-equilibrated PEG and PEO solutions. Scale bars in **(B**,**E**,**H**,**K)** are 50 μm. Scale bars in **(C**,**F**,**I**,**L)** are 500 μm.

ATPSs readily formed from mixtures of PEG and PEO with BSA (Figure 1). As expected, increasing the PEG/PEO MW and concentration favored phase separation with BSA. Thus, PEG 35 kDa and PEO 200 kDa required higher concentrations to achieve phase separation with BSA as compared to PEO 900 kDa and PEO 4,000 kDa. Binodal data were fit according to

$$\begin{array}{l} y=a\exp\left(bx^{0.5}-cx^{3}\right), \end{array}$$

where y and x are the polymer concentrations (in weight percentage), and a, b and c represent fitting parameters (Merchuk et al., 1998). Fitting parameters are displayed for each binodal in Figure 1. Consistent with previous observations, as the polymer MW increased (thus increasing the MW difference between PEG/PEO and BSA), the binodal curves became increasingly asymmetric. Figures 1A,D,G,J also show two coordinates (points 1 and 2) that represent the total compositions of both phases. Figures 1B,E,H,K display the corresponding macroscopic and microscopic images after equilibration of the phases. In Figure 1A, point 1 corresponds to a system comprised of 7% PEG and 15% BSA, and point 2 corresponds to a system comprised of 5% PEG and 20% BSA. Comparing the systems formed from points 1 and 2, one can appreciate that the relative volume of the bottom phase is greater for point 2, as would be predicted from estimation of the tie lines. Although systems on the same tie line have different total polymer concentration and different volume ratios after mixing, they should have the same final polymer concentrations in the top and bottom phases (Kaul, 2000).

Microscopically, one can observe formation of droplet structures for supercritical concentrations of solutes, similar to oil-in-water emulsions. In an ATPS-emulsion, the phase with the greatest volume fraction forms the continuous phase. For instance, in the system composed of 5% PEG and 20% BSA (Figure 1B), the continuous phase is BSA with PEG droplets dispersed within it. Although the interfacial tension of these ATPSs is orders of magnitude lower than oil-in-water systems, the formation of droplets indicates the presence of considerable interfacial tension between the two aqueous phases (Esquena, 2016).

Increasing the molecular weight and concentration of PEG/PEO increased the viscosities of the systems, resulting in longer settling times. The relatively high viscosities of the PEO 200 kDa, PEO 900 kDa, and PEO 4,000 kDa systems resulted in highly deformed microdroplets present in the emulsions (Figures 1E,H,K). The interfacial tension between the phases coupled with their viscosity can enable confinement of cells as well as select biomolecules by way of biomolecular partitioning. However, solutions of PEO 900 kDa above 1.5% and PEO 4,000 kDa above 0.7% are too viscous to experimentally analyze phase separation with BSA and display handling properties that preclude downstream applications. High viscosities of these polymer solutions make pipetting difficult, leading to inaccurate dispensed volumes. This is evident in the images of dispensed droplets of BSA-rich bottom phase into the top PEG-rich phase in Figures 1C,F,I,L. Systems composed of PEG 35 kDa, PEO 200 kDa, and PEO 900 kDa offer workable viscosities that allow dispensing of BSA microdroplets into a continuous phase of PEG/PEO.

**Figure 2:**
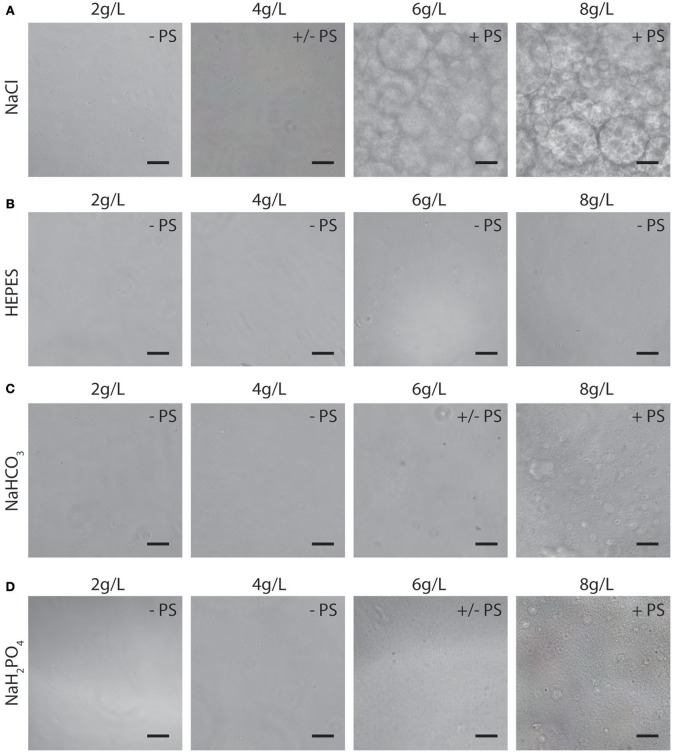
Representative images (10X) of microdroplet emulsions in PEO 900 kDa-BSA systems with addition of NaCl **(A)**, HEPES **(B)**, NaHCO_3_ **(C)**, NAH_2_PO_4_ **(D)**. PS: no phase separation; +/– PS: near critical system/slight phase separation; + PS: super-critical system with apparent phase separation. Scale bars are 50 μm.

In terms of ATPS thermodynamics, the free energy of mixing must be positive for phase separation to occur, which means that the enthalpy of mixing must dominate over the entropy of mixing (Frith, 2010). The addition of certain salts to an ATPS is thought to decrease the entropic penalty, thereby promoting de-mixing of the system at lower polymer concentrations (Frith, 2010; Johansson et al., 2011). We reasoned that salts and buffering compounds present in various cell culture medium formulations would therefore influence phase separation of PEG/PEO-BSA ATPSs. The influence of salts such as NaCl and buffering compounds such as HEPES, NaHCO_3_ and NaH_2_PO_4_ on phase separation was therefore examined for a single ATPS formulation without extensive characterization of binodal phase diagrams (Figure 2). As shown in Figure 2A, increasing NaCl concentration promoted ATPS formation. Concentrations of NaCl in cell culture medium range from 4.5 to 7.6 g/L. Therefore, the NaCl concentration of a typical cell culture medium formulation is sufficient to promote phase separation of PEG/PEO and BSA. In contrast, HEPES, a buffering compound present in Iscove's Modified Dulbecco's Media (IMDM) at ~6 g/L did not promote ATPS formation (Figure 2B). Sodium bicarbonate and sodium phosphate monobasic (also present in various medium formulations at concentrations ranging from ~1.2 to 3.7 g/L, and ~0.100 to 0.580 g/L, respectively) had modest effects on ATPS formation as shown in Figures 2C,D. The type of salt added to the system also influenced the shape and size of the microdroplets. Microdroplet emulsions formed in systems containing NaCl displayed droplets that were more well-defined and rounder than droplets formed in systems containing sodium bicarbonate and sodium phosphate monobasic, indicating that addition of NaCl influences the interfacial tension by strongly favoring de-mixing of the polymers.

**Figure 3:**
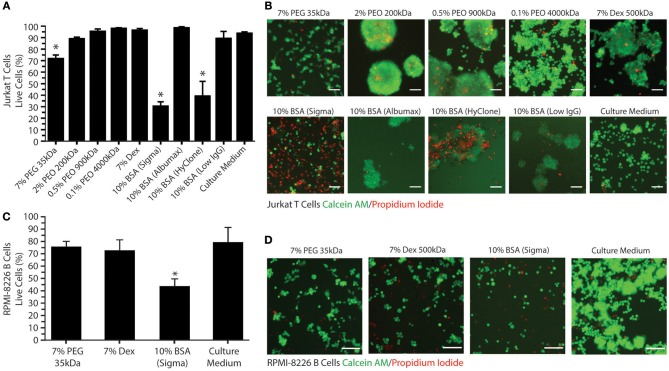
Jurkat T cell and RPMI-8226 B cell viability in ATPS-polymer solutions. **(A)** Percentage of viable Jurkat T cells present for various polymer/BSA medium additives after 72 h. **(B)** Representative images (10X) of live Jurkat T cells (in green, stained with C-AM) and dead cells (in red, stained with PI) for various polymer/BSA medium additives after 72 h. **(C)** Percentage of viable RPMI-8226 B cells present for various polymer/BSA medium additives after 72 h. **(D)** Representative images (10X) of live RPMI-8226 B cells (in green) and dead cells (in red) for various polymer/BSA medium additives after 72 h. Scale bars are 50 μm. Significant reductions in cell viability relative to the control are indicated by *.

Having characterized the phase separation of this novel system, we next sought to determine the effects that PEG/PEO and BSA had on cell viability and function. We evaluated the viability of Jurkat T cells in individual phase-separating solutions present in the PEG/PEO-BSA and PEG-Dex systems to assess the suitability of these polymers for long term cell culture. As shown in Figures 3A,B, culturing Jurkat T cells for 72 h in 7% PEG, resulted in ~70% cell viability, as compared to >89% viability when the cells were cultured in the three types of PEO tested. Among the PEOs, 0.1% PEO 4,000 kDa had the least impact on cell viability with ~98% cell viability. Therefore, polymer concentration has a considerable effect on cell viability over time. Addition of either technical grade BSA from Sigma or HyClone BSA to the medium resulted in <40% cell viability. However, addition of cell culture grade Albumax BSA to the medium resulted in 98% cell viability. Addition of low IgG BSA to the medium resulted in ~90% cell viability. Cells cultured in Dex were ~97% viable. We also evaluated the viability of RPMI-8226 B cells in a selection of individual phase-separating solutions to confirm the results obtained for T cells using an additional suspension-cultured immune cell line (Figures 3C,D). Culturing RPMI B cells for 72 h in 7% PEG 35kDa resulted in ~76% cell viability. The 7% Dex solution had a similar impact on B cell viability with ~73% cell viability. Cells cultured in the technical grade BSA (Sigma) were ~44% viable, which was significantly different compared to control (~80% cell viability). Since the impact of Sigma BSA on RPMI-8226 B cell viability was slightly lower in comparison with Jurkat T cells, we presumed that the cell culture grade Albumax BSA would not affect B cell viability. These results suggest that both PEG-BSA and PEG-Dex systems are suitable for culture of T cells and B over 72 h.

**Figure 4:**
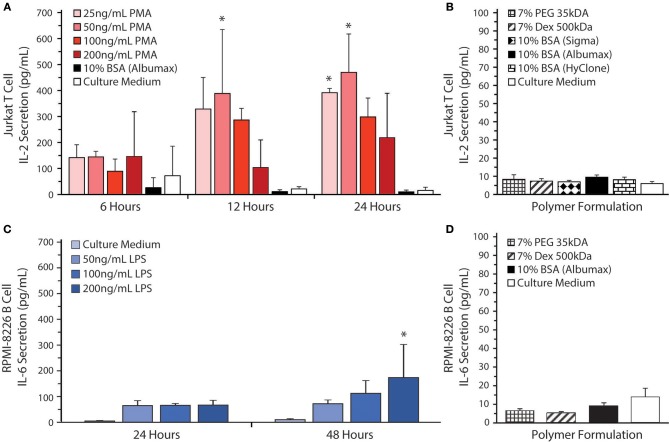
Cytokine secretion by Jurkat T cells and RPMI-8226 B cells. **(A)** IL-2 levels were measured by ELISA in culture supernatants from Jurkat T cells at 6, 12, and 24 h after stimulation with 25, 50, 100, or 200 ng/mL of PMA in RPMI 1640 medium as a positive control for cell stimulation. All the stimulation conditions also received 300 ng/mL of ionomycin. The IL-2 secretion by Jurkat T-cells was also measured when the cells were cultured in 10% BSA (Albumax) without stimulation. RPMI 1640 medium was used as a negative control. Significant differences in cell activation relative to the control are indicated by *. **(B)** Jurkat T cells were cultured in 10% BSA (Albumax, Sigma, or HyClone) 7 % Dex, and 7% PEG. RPMI 1640 medium was used as a control. **(C)** IL-6 levels were measured by ELISA in culture supernatants from RPMI-8226 B cells at 24 and 48 h after stimulation with 50, 100, or 200 ng/mL of LPS in RPMI 1640 medium as a positive control for cell stimulation. RPMI 1640 medium without LPS was used as a negative control. Significant differences in cell activation relative to the control are indicated by *. **(D)** RPMI-8226 B cells were cultured in 10% BSA (Albumax) 7% Dex and 7% PEG 35 kDa. Both Jurkat T cell and RPMI-8226 B cell stimulation in various medium additives remained near baseline with no significant differences noted between polymer conditions and the culture medium control.

All polymer solutions examined tended to cause T cell aggregation, as shown in Figure 3B. Aggregation was most evident in the PEO, BSA, and Dex conditions. This effect can result from either excessive cell growth or immune cell activation. Therefore, we decided to investigate the effects of the phase-separating solutions on cell activation by measuring IL-2 secretion from Jurkat T cells and IL-6 secretion from RPMI-8226 B cells as these are the main cytokines secreted by these cells upon activation. As a positive control for T cell activation, Jurkat T cells were treated with PMA and ionomycin (Figure 4A). To find the optimal PMA concentration to activate the cells, we performed a dose-response experiment with PMA concentrations ranging from 25 to 200 ng/mL. There were no significant differences between the four different PMA concentrations tested over the first 6 h. After 12 h of exposure, 50 ng/mL PMA resulted in the highest level of IL-2 secretion out of any of the concentrations tested (~390 pg/mL IL-2), reaching a peak of ~470 pg/mL IL-2 after 24 h. As shown in Figure 4B, the polymers themselves do not stimulate IL-2 secretion over 24 h. Activation of Jurkat T cells by PMA/ionomycin treatment in polymer/BSA-containing medium was also confirmed. Thus, although Albumax, HyClone, and Dex cause cell aggregation, they do not stimulate IL-2 secretion and are therefore unlikely to have significant effects on cell activation.

To confirm that the polymer solutions did not stimulate RPMI-8626 B cells, we measured IL-6 secretion. As a positive control for B cell activation, RPMI-8226 B cells were treated with LPS (Figure 4C). To find the optimal LPS concentration to activate the cells, we performed a dose-response experiment with LPS concentrations ranging from 50 to 200 ng/mL. There were no significant differences between the four different LPS concentrations tested over the first 24 h. After 48 h of exposure, however, 200 ng/mL LPS resulted in the highest level of IL-6 secretion out of any of the concentrations tested (~175 pg/mL IL-6) and was significantly different from the control (~9.5 pg/mL IL-6). As shown in Figure 4D, the polymers themselves do not stimulate IL-6 secretion. Thus, Albumax, PEG, and Dex solutions do not stimulate IL-6 secretion and are therefore unlikely to have significant effects on cell activation, although additional cytokines should be examined in the future to confirm this observation.

**Figure 5:**
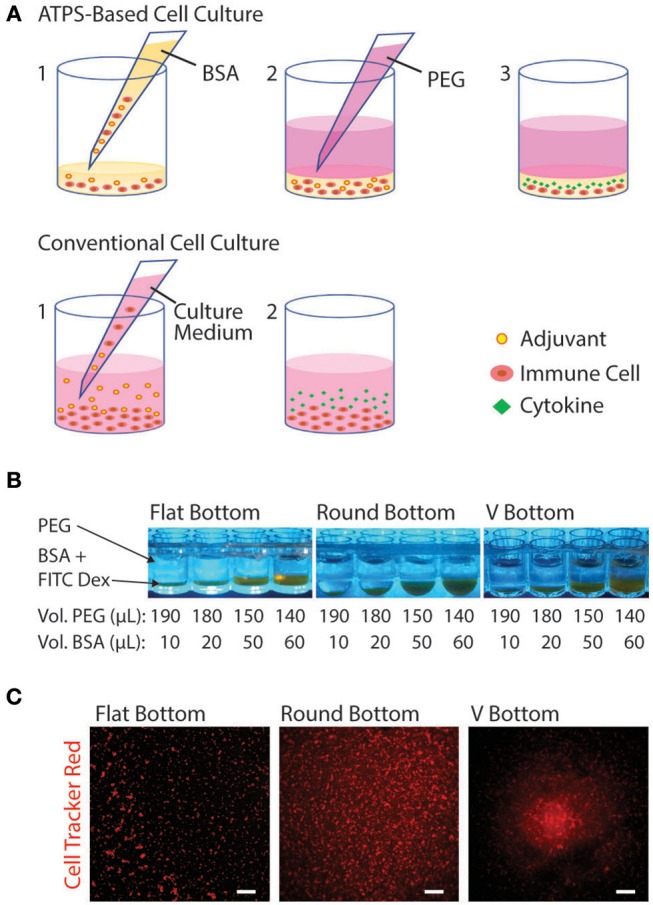
Schematic diagram of the PEG-BSA system for compartmentalized immune cell culture. **(A)** Experiment setup using ATPS: (1) Immune cells are suspended in the BSA solution containing reagents (e.g., adjuvants), and 20 μL of the mixture is added to each well of a 96-well plate. (2) An 80 μL volume of the PEG solution is added to the well to cover the BSA-bottom phase, confining cells and reagents in the BSA solution. (3) Over 24 h of adjuvant stimulation, immune cells produce cytokines, which remain confined in the bottom phase. Conventional experiment setup: (1) Immune cells and reagents are mixed in culture medium and added to each well. (2) Over 24 h of stimulation, immune cells secrete cytokines that diffuse throughout the culture medium. **(B)** PEG-BSA systems in three 96-well plate geometries: flat-bottom, round bottom, and V bottom. The BSA-phase (bottom) is labeled with FITC-Dex. **(C)** Representative images (4X) of Jurkat T cells cultured in the PEG-BSA system in flat-bottom, round-bottom, and V-bottom 96-well plates. The cells were stained with CellTracker Red™ CMTPX. Scale bars are 100 μm.

Due to the mild environment provided by ATPSs, many applications have been developed in cell biology, e.g., for cell purification and more recently for micropatterning of cells for tissue engineering and high-throughput drug screening (Tavana and Takayama, 2011; Atefi et al., 2014; Agarwal et al., 2017). These techniques mainly use the well-known PEG-Dex system to specifically confine cells to one of the phases or to the interface between the phases (Tavana et al., 2011; Frampton et al., 2015). Previous work has focused on applications using adherent cells where the polymers are applied acutely to confine the cells and are subsequently washed away once the cells attach or assemble into aggregates. For applications involving suspension-cultured cells such as immune cells, however, the phase-separating solutions must remain in place to confine the cells over longer periods of time. Figure 5A shows a schematic diagram comparing ATPS-based immune cell culture with the conventional cell culture. The PEG-BSA system can potentially be applied for confining immune cells to reduce cell and reagent consumption in screening assays (e.g., adjuvants and antigens for vaccine development). For ATPS-based cell culture, immune cells and reagents are mixed in a BSA solution and covered with a PEG solution. Over 24 h of stimulation, immune cells produce cytokines, which remain confined in the bottom phase. For conventional cell culture, immune cells, and reagents such as adjuvants are mixed in culture medium only. Over 24 h of stimulation, immune cells secrete cytokines that diffuse throughout the culture medium.

To begin optimizing this system, we compared the confinement of immune cells in 96-well plates with three distinct well bottom shapes: flat-bottom, round-bottom, and V-bottom. In addition, we added different volume ratios of PEG:BSA (10:190, 20:180, 50:150, 60:140) (Figure 5B). Both variables (plate type and ATPS volume ratio) were evaluated in terms of cell confinement and ability to perform microscopic imaging. To better visualize the ATPS phases and the cells, the BSA phase was labeled with FITC-Dex and the Jurkat T cells were stained with CellTracker Red™ CMTPX. As shown in Figure 5B, both PEG and BSA phases can be easily visualized in each well type. In terms of cell confinement, the V-bottom well was most effective at localizing cells to a central well region, as shown in Figure 5C, with the effect of the ATPS mainly to prevent cell disruption during medium changes and supernatant collection. All four volume ratios enabled confinement and imaging of cells. Thus, the 10:190 volume ratio is the most advantageous volume ratio for minimizing cell and reagent consumption.

**Figure 6:**
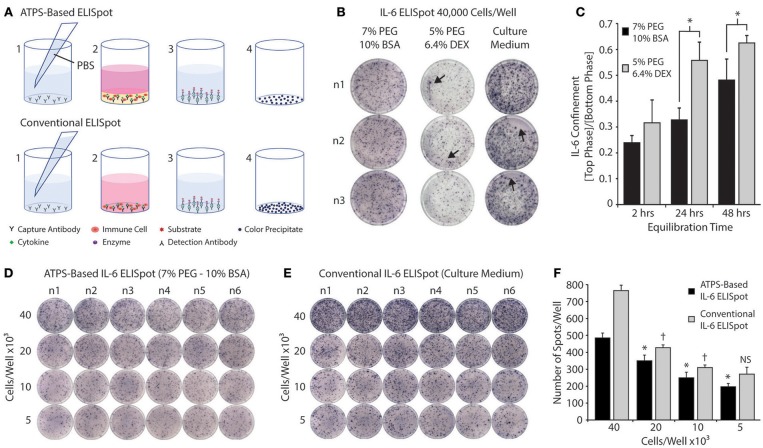
Screening immune cells in the PEG-BSA system by ELISpot. **(A)** ATPS-Based ELISpot: (1) Capture antibodies are diluted in PBS and added to each well. After overnight incubation, the capture antibodies are washed away and replaced with immune cells and adjuvants mixed in the BSA-phase. Over 24 h of stimulation, cytokines are produced by the immune cells and become confined in the BSA-phase where they bind to the capture antibodies. (3) The PEG-BSA system is washed away and it is replaced with detection antibodies mixed in PBS, followed by streptavidin-alkaline phosphatase and substrate solutions. (4) Each colored spot detected on the membrane at the bottom of each well corresponds to a cytokine-secreting cell. Conventional ELISpot: (1) Capture antibodies are mixed in PBS and added to each well of an ELISpot plate. After overnight incubation, the capture antibodies are washed away and replaced with immune cells suspended in culture medium containing adjuvants. Over 24 h of stimulation, the cytokines produced by immune cells bind to capture antibodies. (3) The culture medium is washed away and it is replaced with detection antibodies mixed in PBS, followed by enzyme and substrate solutions. (4) Each colored spot detected on the membrane at the bottom of each well corresponds to a cytokine-secreting cell. **(B)** Representative ELISpot results for RPMI-8226 B cells stimulated with LPS and incubated in 7% PEG-10% BSA, 5% PEG-6.4% Dex, and culture medium for 24 h. Artifacts are indicated by arrows pointing to line artifacts from cell movement, ring artifacts along the well edges, and regions of the well devoid of spots. **(C)** IL-6 confinement in the bulk phases of two ATPS formulations over time: 10%BSA-7%PEG and 6.4%Dex-5%PEG. * p < 0.05 (ANOVA with Tukey *post-hoc* test). **(D)** Representative ELISpot results for RPMI-8226 B cells stimulated with LPS and incubated for 24 h in the 7% PEG-10% BSA system. **(E)** Representative ELISpot results for RPMI-8226 B cells stimulated with LPS and incubated for 24 h in culture medium (conventional method). **(F)** The spots formed within wells using both methods were counted and plotted as a function of cell density. The * denotes significant differences from the next highest cell density for the ATPS-based method. The †denotes significant differences from the next highest cell density for the conventional method. NS denotes no significant difference from the next highest cell density.

In order to evaluate the potential of the PEG-BSA system for screening vaccine adjuvants *in vitro*, we conducted an ATPS-based ELISpot using the PEG-BSA system. Figure 6A shows a schematic diagram comparing the ATPS-based ELISpot with the conventional ELISpot assay. Both procedures follow the same protocol, except that the ATPS-based ELISpot confines cells and cytokines in the bottom BSA phase. Figure 6B compares ELISpot results obtained from the PEG-BSA system with those obtained from the PEG-Dex system and the conventional assay system. The PEG-BSA system produced spots that were qualitatively similar to those produced using the conventional assay protocol. Artifacts were more frequently observed for the PEG-Dex and conventional assay than in the PEG-BSA assay, as indicated by arrows pointing to line artifacts from cell movement, ring artifacts along the well edges and regions of the well devoid of spots. When the ELISpot assay was conducted using the PEG-Dex system to confine the cells, the overall signals and quality of the spots were appreciably diminished compared to the PEG-BSA and conventional assay formats.

We also examined IL-6 confinement in the 7% PEG-10% BSA and 5% PEG-6.4% Dex systems by adding 150 μL of the top phase (7% PEG or 5% PEG) on top of 50 μL of the bottom phase (10% BSA or 6.4% Dex), with the bottom phase spiked with recombinant IL-6 (500 pg/mL). After thermodynamic equilibration at room temperature, we collected each phase separately for analysis, measured the IL-6 concentration in each phase and plotted the ratio of IL-6 concentration between the top phase and bottom phase. As shown in Figure 6C, IL-6 was more strongly confined in the BSA phase of the PEG-BSA system than in the Dex phase of the PEG-Dex system, suggesting that the PEG-BSA system is superior to the PEG-Dex phase system for confining cytokines in the ELISpot assay and in other immunoassays used for screening cell responses. This may also partially explain why the quality of the spots in the ELISpot assay were better for the PEG-BSA system as compared to the PEG-Dex system.

Finally, Figures 6D,E compares images of ATPS-based ELISpot using the PEG-BSA system and conventional ELISpot as a function of cell seeding density. Compared to the conventional technique, ATPS-based ELISpot presented less background noise and a lower frequency of spot development. As shown in Figure 6F, the number of spots developed using the ATPS-based ELISpot is smaller in comparison with the conventional assay across different cell densities. However, there is a significant difference in spot number between ATPS-based ELISpot and conventional ELISpot assay for each cell density. Interestingly, using the ATPS-based assay there is a significant difference between each cell density, whereas with the conventional method, there is no significant difference between 10 × 10^3^ and 5 × 10^3^ cells per well, suggesting that the ATPS technique may improve assay sensitivity when measuring low numbers of cytokine secreting cells.

## Discussion

Microscale technologies are frequently used to investigate cell-material interactions (Anderson et al., 2005; Khetani and Bhatia, 2008), engineer and control cell shape and function (Singhvi et al., 1994), and evaluate cell responses (Chen et al., 2005) in a high-throughput manner. These technologies offer tight control of the cellular microenvironment, significantly reduce the consumption of cells and reagents required to perform experiments, and in some cases, improve reaction efficiencies (Chung et al., 2007). Many of these platforms rely on microfluidics to manipulate the small volumes of fluid used for cell culture and analysis. Microfluidic array platforms have been developed for high-throughput cytotoxicity screening, where three types of mammalian cell lines were screened against low and high concentrations of five toxins (digitonin, saponin, CoCl_2_, NiCl_2_, and acrolein) (Wang et al., 2007). Another example is the sophisticated system developed by Choi and Cunningham, which integrates a 96-well microplate with microfluidic networks and biosensors to measure the binding of IgG molecules (20 μL IgG solution consumed) to immobilized protein A (30 μL protein A solution consumed) (Choi and Cunningham, 2007). Although both systems are able to analyze cell viability and biomolecular interactions in a multiplex format while minimizing reagent consumption, the specialized technical expertise and equipment required to operate systems such as these limits their utility in life science laboratories.

In contrast to Dex, BSA is ionic and amphiphilic, which may intensify BSA interactions with cells and biomolecules. Our findings suggest that the PEG-BSA system has potential utility in confining immune cells in the bottom phase without significantly affecting cellular viability and activation. In addition, the BSA phase promotes superior IL-6 confinement over time compared to the Dex phase which may be advantageous for detection by various immunoassays such as ELISpot. In terms of practical application, our ATPS-based multiplex screening does not rely on expensive and complex equipment and can be easily adapted to any laboratory familiar with commonly-available liquid handing tools such as handheld micropipettes. The approach presented here can micropattern immune cells in an ATPS, while retaining high cell viability and minimizing cell activation as measured by IL-2 and IL-6 secretion.

There is considerable interest in polymers that are naturally immunostimulatory. Short cationic amphiphilic peptides are produced by several different cell types and can modulate the innate immune response, which makes them useful as adjuvants (Hancock et al., 2016). Polysaccharides also stimulate immune responses and have been used as adjuvants. For example, inulin-based adjuvants have been shown to activate both humoral and cellular immune responses (Petrovsky, 2006; Kumar et al., 2016). Chitosan is another polysaccharide that elicits a cellular immune response (Moran et al., 2018). We anticipate that our system may be used to confine various biomolecules and immunostimulatory polymers and particles within the BSA phase via biomolecular partitioning for future applications in screening novel immunotherapies and adjuvant formulations. In this future work, it will be important to consider the handling properties of the ATPS-forming solutions and to more completely characterize the phenotype of cells used in experiments by assessing other types of immune cells and secreted cytokines. This cell characterization work will be particularly important in light of the cell aggregation phenomenon we observed for Jurkat T cells, since cell-cell interactions are functionally important for T cell signaling, as they are for other immune cells (Sabatos et al., 2008). However, our data for IL-2 secretion suggest that aggregation of Jurkat T cells cultured in ATPSs does not necessarily correlate with cell activation.

Although most ATPSs used in cell culture applications have been used to biopattern adherent cells (Tavana et al., 2009; Frampton et al., 2013), ATPS techniques have also been developed to form non-adherent cell aggregates. By exploiting the interfacial forces between PEG and Dex, Frampton et al. developed a simple method to confine cells and promote their assembly into tissue constructs (Frampton et al., 2015). Initially, cells were suspended in the PEG phase, which was layered on top of the Dex phase. The cells were then allowed to settle by gravity to the PEG-Dex interface. Once at the interface, cell-cell connections were formed, leading to the self-assembly of constructs over several hours. ATPS techniques have also enabled generation of tumor spheroids by confining cells within a nanoliter-volume droplet of Dex immersed in a PEG phase (Atefi et al., 2014; Han et al., 2015). However, to the best of our knowledge, an ATPS has not been used to confine suspension-cultured cells such as immune cells. Confining suspension-cultured cells in an ATPS can be more challenging than adherent cells because the polymers must remain in place to continue to confine the cells.

A novel application for the PEG-BSA system is in confinement of immune cell and reagents such as adjuvants and antigens used in vaccine formulation development. ELISpot is a technique widely used for screening vaccine candidates by measuring cellular immune responses to a specific stimulus (Chambers et al., 2010). The ATPS-based ELISpot was used to demonstrate the application of the PEG-BSA system to confine immune cells to facilitate vaccine adjuvant screening *in vitro*. When conducting ELISpot using BSA as the bottom phase, the BSA solution reduced spot development by blocking non-specific binding and consequently improving the sensitivity of the assay. One can notice a significant difference between each cell density using the PEG-BSA system to confine cells and cytokines. However, with the conventional method, there is no significant difference between the lowest cell densities (10 × 10^3^ and 5 × 10^3^ cells/well), which means that the ATPS-based ELISpot has potential to improve the sensitivity of this assay. Addressing how additional adjuvants and cytokines other than IL-6 partition between the PEG and BSA phase will be of paramount importance in determining the broad utility of this approach.

Finally, the ATPS-based approach described here opens up possibilities for applications beyond the culture of immune cell lines for screening applications. For example, mixtures of adherent and non-adherent cell types can potentially be co-cultured in this platform to investigate cell interactions and better understand the complexity of immune cell signaling in various tissues. Furthermore, the PEG-BSA system has potential to be applied to separate and purify biomolecules according to their affinity for each phase. The PEG/PEO-BSA system may also find applications in the encapsulation of drugs and biomolecules. Thus, characterization of this novel ATPS and preliminary evaluation of the cytotoxicity and cellular responses to the phase-forming solutes may be valuable for exploration of a variety of future biomedical applications.

## Conclusion

We characterized a new ATPS and demonstrated that immune cells and cytokines can be confined in a PEG-BSA system without compromising cell viability and activation. We showed that this new system can be employed to improve the sensitivity of a conventional ELISpot assay by reducing background noise and detecting differences between small numbers of cytokine secreting cells. We anticipate that this new system will enable more robust and cost-effective multiplex screening of adjuvants and other compounds known to stimulate immune cells.

## Author Contributions

AT performed or assisted with all experiments and wrote the paper. AK performed the phase-separation characterization and helped write the paper. RA helped analyze data and edited the paper. NT helped analyze data and edited the paper. JW contributed to the initial concept of this work and assisted with experimental planning. JF oversaw all experiments and edited the paper.

### Conflict of Interest Statement

The authors declare that the research was conducted in the absence of any commercial or financial relationships that could be construed as a potential conflict of interest.
