# Corrigendum: Confinement of Suspension-Cultured Cells in Polyethylene Glycol/Polyethylene Oxide-Albumin Aqueous Two-Phase Systems

**DOI:** 10.3389/fchem.2019.00670

**Published:** 2019-10-04

**Authors:** Alyne G. Teixeira, Alex Kleinman, Rishima Agarwal, Nicky W. Tam, Jun Wang, John P. Frampton

**Affiliations:** ^1^School of Biomedical Engineering, Dalhousie University, Halifax, NS, Canada; ^2^Pomona College, Claremont, CA, United States; ^3^Department of Microbiology and Immunology, Dalhousie University, Halifax, NS, Canada; ^4^Canadian Center for Vaccinology, IWK Health Centre, Halifax, NS, Canada

**Keywords:** aqueous two-phase systems, cell partitioning, polyethylene glycol, polyethylene oxide, bovine serum albumin, immune cells, ELISpot

**Jun Wang** was not included as an author in the published article. The corrected Author Contributions Statement appears below:

“AT performed or assisted with all experiments and wrote the paper. AK performed the phase-separation characterization and helped write the paper. RA helped analyze data and edited the paper. NT helped analyze data and edited the paper. JW contributed to the initial concept of this work and assisted with experimental planning. JF oversaw all experiments and edited the paper.”

The authors apologize for this error and state that this does not change the scientific conclusions of the article in any way. The original article has been updated.

